# Fucosylated inhibitors of recently identified bangle lectin from *Photorhabdus asymbiotica*

**DOI:** 10.1038/s41598-019-51357-9

**Published:** 2019-10-17

**Authors:** Gita Paulíková, Josef Houser, Martina Kašáková, Beáta Oroszová, Benedetta Bertolotti, Kamil Parkan, Jitka Moravcová, Michaela Wimmerová

**Affiliations:** 1 Central European Institute of Technology, Masaryk University, Brno, Czech Republic; 2 National Centre for Biomolecular Research, Faculty of Science, Masaryk University, Brno, Czech Republic; 3 Department of Chemistry of Natural Compounds, University of Chemistry and Technology, Prague, Czech Republic; 4 Department of Biochemistry, Faculty of Science, Masaryk University, Brno, Czech Republic

**Keywords:** Glycobiology, X-ray crystallography

## Abstract

A recently described bangle lectin (PHL) from the bacterium *Photorhabdus asymbiotica* was identified as a mainly fucose-binding protein that could play an important role in the host-pathogen interaction and in the modulation of host immune response. Structural studies showed that PHL is a homo-dimer that contains up to seven l-fucose-specific binding sites per monomer. For these reasons, potential ligands of the PHL lectin: α-l-fucopyranosyl-containing mono-, di-, tetra-, hexa- and dodecavalent ligands were tested. Two types of polyvalent structures were investigated – calix[4]arenes and dendrimers. The shared feature of all these structures was a *C*-glycosidic bond instead of the more common but physiologically unstable *O*-glycosidic bond. The inhibition potential of the tested structures was assessed using different techniques – hemagglutination, surface plasmon resonance, isothermal titration calorimetry, and cell cross-linking. All the ligands proved to be better than free l-fucose. The most active hexavalent dendrimer exhibited affinity three orders of magnitude higher than that of standard l-fucose. To determine the binding mode of some ligands, crystal complex PHL/fucosides **2** – **4** were prepared and studied using X-ray crystallography. The electron density in complexes proved the presence of the compounds in 6 out of 7 fucose-binding sites.

## Introduction

Reversible interactions between the oligosaccharide chain of glycolipids/glycoproteins and carbohydrate-binding protein receptors (lectins) mediate critical biological recognition at the cell level. They are of vital importance in many normal and pathological processes ranging from fertilization, immune response and intercellular communication to viral/bacterial invasion and tumour metastasis^1^. The identification of lectin-carbohydrate interactions in human diseases raises the possibility of the development of carbohydrate-based therapeutic products. Anti-adhesion therapy has been used for a few decades and the protective effect of anti-adhesive saccharides has been demonstrated in several studies^2^. For example, mannose has been used to treat urinary tract infections caused by *E. coli*. Moreover, the anti-adhesive agents do not kill pathogens or arrest their growth thus development of resistance to anti-adhesive agents will be less likely as compared to antibiotics^2,3^. Therefore, anti-adhesion therapy is a very attractive approach that not only prevents infections at an early stage but is also an important alternative treatment for bacterial infection considering the alarming increase of drug-resistant bacteria.

*P. asymbiotica* was originally considered to be exclusively an insect pathogen until 1989, when it was isolated from a patient in the USA for the first time^4^. Later on, further human infections were reported in Australia^5^ and nowadays, occurrence of this bacterium is not restricted to American and Australian continents but starts to be globally spread^6–10^. *P. asymbiotica* is regarded as an emerging human pathogen causing either locally invasive or disseminated soft-tissue infections^11–14^. Relatively low number of infections has been reported so far, however this is likely to be caused by misidentification by automated microbiological techniques, which do not contain *Photorhabdus* sp. in their databases^12^. Recently, the two subspecies of *P. asymbiotica* (*P. asymbiotica* subsp. *asymbiotica* and *P. asymbiotica* subsp. *australis*) were suggested to be elevated into a higher taxon, creating the *P. asymbiotica* and *P. australis* species, respectively^15^.

As a part of our ongoing programme focused on host-pathogen interactions at the molecular level, we identified a lectin in the *P. asymbiotica* genome designated PHL, which we prepared in a fully functional recombinant form^16^. We also determined its carbohydrate specificity and investigated the structural details of ligand binding. Moreover, PHL demonstrated the ability to act as a host-cell recognizing agent through interacting with the haemolymph of *Galleria mellonella* (order Lepidoptera, family Pyralidae) and human blood components and also to modulate immune system of both hosts^16^. Lectins of other bacteria were confirmed as virulence factors, e.g. PA-IL and PA-IIL from opportunistic *Pseudomonas aeruginosa* or FimH from uropathogenic *Escherichia coli* (UPEC) strains^17,18^. The PHL lectin can play equally important role as above-mentioned examples and therefore, PHL could be a usable therapeutic target. Interestingly, other two saccharide-binding proteins were detected in *Photorhabuds* genus – PLL and PllA, both from *P. luminescens*^19,20^. PLL is a fucose-specific lectin and a sequence homologue of PHL. However, PLL is not able to modulate the host innate immune response as PHL and probably plays a role in bacteria-nematode interactions. The PllA lectin is a galactose-specific lectin and homologue of PA-IL (LecA) from *Pseudomonas aeruginosa*, which is known for its utilization in *P. aeruginosa* infection and biofilm formation in lung of patients with cystic fibrosis^21^. Due to the PllA specificity, the certain role both in the symbiosis with their nematode hosts and in insect infection is speculated.

The lectin PHL interacts selectively, mainly with l-fucose and oligosaccharides containing terminal l-fucose residue, and prefers an α-glycosidic bond over a β-linkage. Surface plasmon resonance (SPR) in a competition assay revealed that methyl α-l-fucopyranoside, with an IC_50_ of 40 μM, was about a 10-fold stronger ligand than free l-fucose. Nevertheless, the IC_50_ value is in the low μM range, suggesting a relatively weak binding of methyl α-l-fucopyranoside to PHL. The preparation of a core l-fucose derivative bearing suitable functional groups for the construction of multivalent systems could enhance this affinity substantially.

Here we present a comprehensive study covering interactions of the lectin PHL with a pool of l-fucose-based potential ligands with different valences and topologies, assessed by hemagglutination, SPR, isothermal titration calorimetry (ITC), and cell cross-linking assays. All constructs are stable against both acidic and enzymatic hydrolysis due to the presence of *C*-glycosidic bonds. In most of the experiments, dendrimers emerged as the best ligands, especially the hexafucosylated dendrimer **9**. During SPR measurement, dendrimer **9** had a potency per fucose unit that was over 1,600 times as effective as l-fucose and 52 times as respective saccharide-building unit azide **1**. The structure of PHL complexes with fucosides **2**–**4** was solved using X-ray crystallography, and compounds were found in 6 out of 7 fucose-binding sites of at least one PHL monomer unit.

## Result and Discussion

### Ligands tested

**Figure 1:**
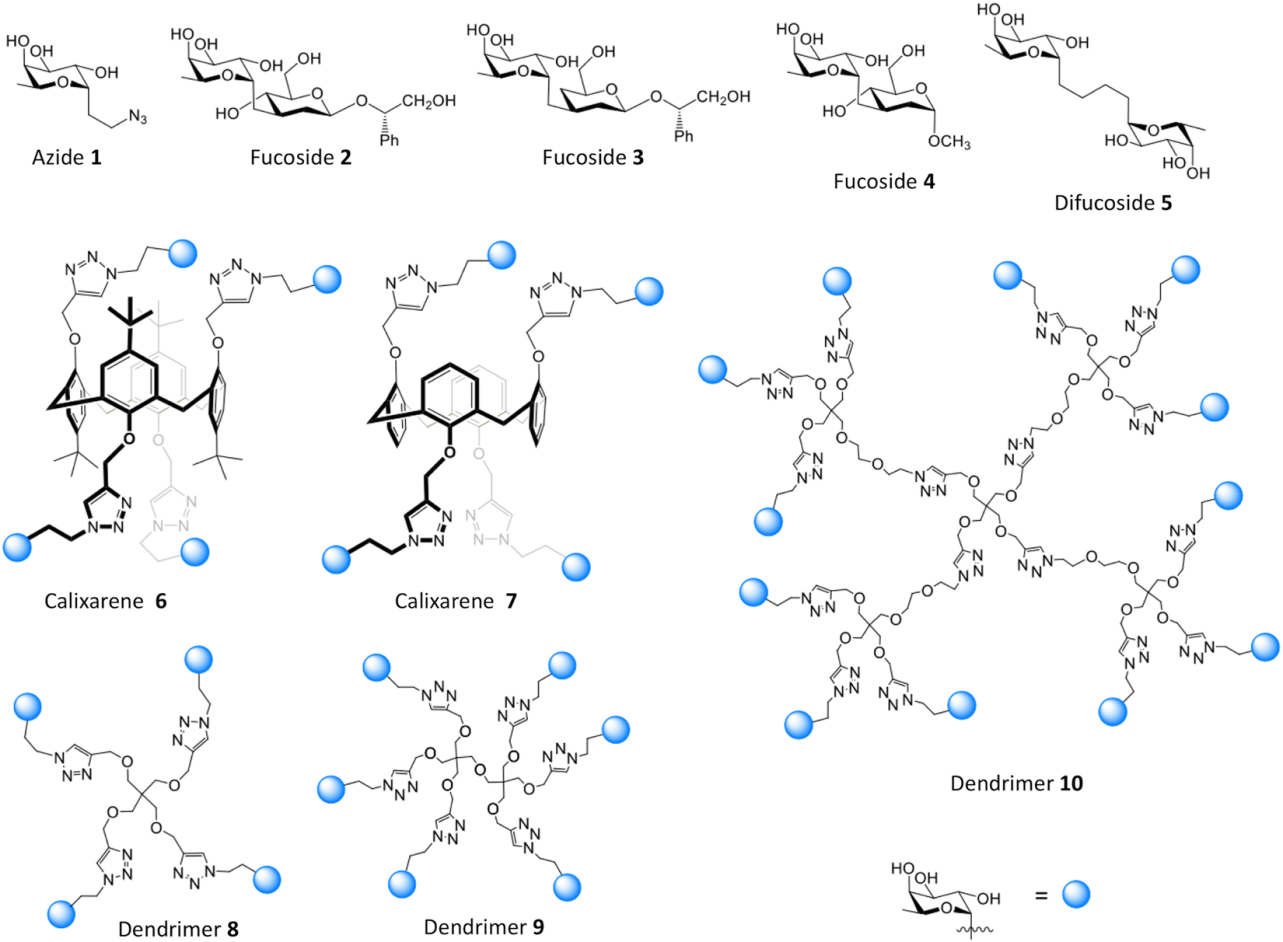
Structure of ten tested α-l-fucopyranosyl compounds.

In total 10 compounds with α-l-fucopyranosyl unit(s) were selected from our library of recently synthesized potential ligands towards various lectins (Fig. 1). Azide **1**^22^ is an analogue of methyl α-l-fucopyranoside, and also serves for the preparation of polyvalent systems. The previous study showed increased affinity to di- and tri-saccharides for PHL. Therefore, pseudo-*C*-disaccharides **2**–**4**^23^ as monovalent ligands differing in the structure of the *C*-aglycone part were included in the study to analyse the potential influence of the second saccharide-like unit on the ligand binding. 1,4-bis(fucopyranosyl)butane **5**^23^ is a divalent mimetic in which the distance between the C1 atoms of both α-l-fucopyranosyl units is derived from the structure of blood group Lewis b tetrasaccharide. Tetravalent constructs **6** and **7** contain a calix[4]arene scaffold in a 1,3-*alternate* conformation that ensures sufficient solubility in water. Both **6** and **7**^24^ were prepared by CuAAC click reaction^25,26^ from azide **1** and the respective tetrapropargyl calix[4]arene. Finally, a set of dendrimers **8**–**10**^22^ with valences ranging from 4 to 12 are more flexible and complex ligands than calix[4]arene derivatives, thus a significant increase in the complexity of the binding event can be expected. The affinity improvements in multivalent carbohydrate−lectin interactions have been attributed to the so-called “cluster glycoside effect”^27–29^.

### Hemagglutination inhibition assay

**Figure 2:**
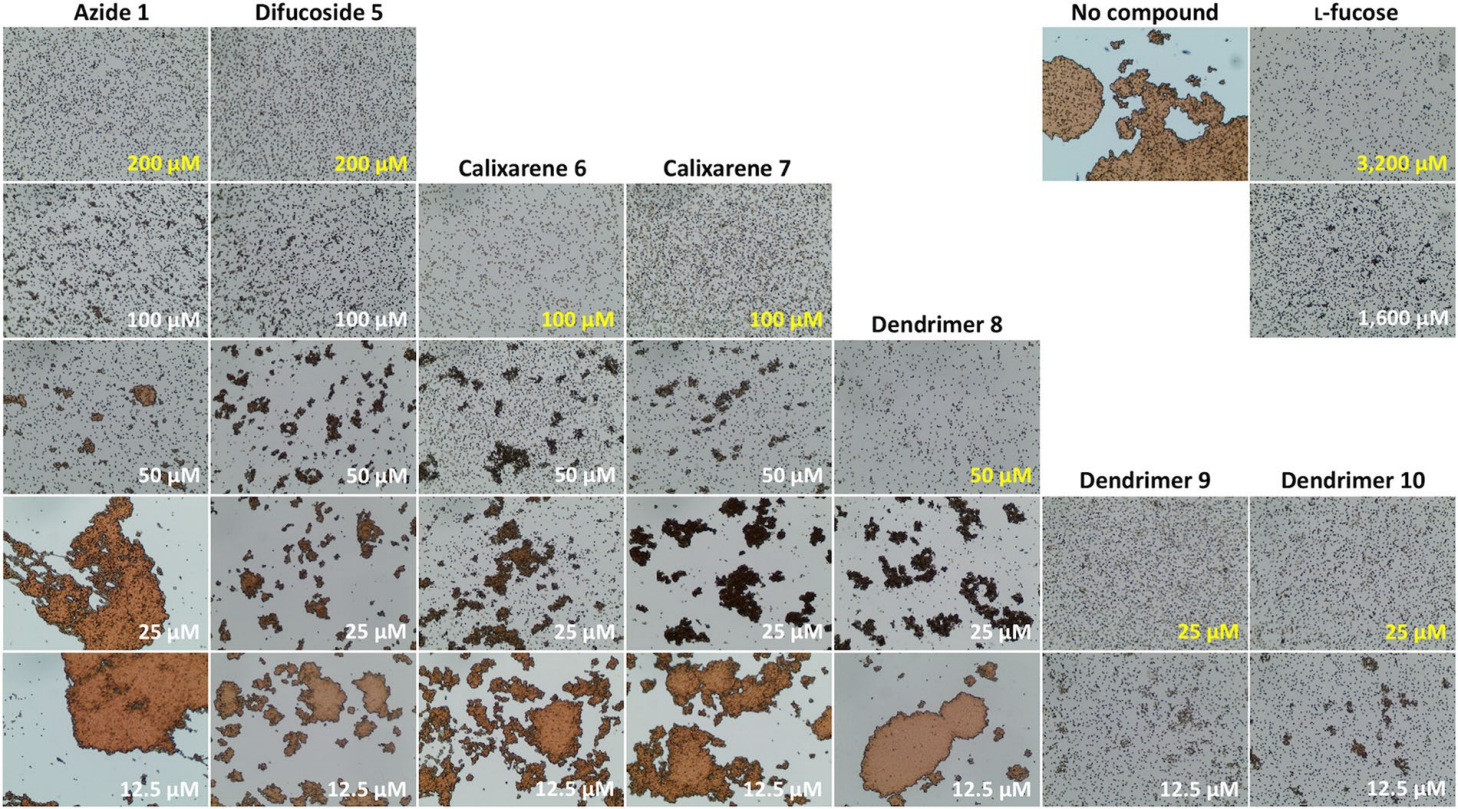
Representatives of α-l-fucopyranosyl compounds with a higher potency than α-Me-Fuc and their influence on hemagglutination of RBC O caused by PHL. Two-fold serially diluted carbohydrate compounds **1, 5**–**10** were able to inhibit hemagglutination at the minimal concentrations highlighted in yellow. The hemagglutination of RBC O caused by PHL without an inhibitor is shown in the picture in the upper right corner.

PHL as a fucose-specific lectin was shown to agglutinate papain-treated RBCs of blood group O^16^ with the surface-exposed terminal trisaccharide α-l-Fuc*p*-(1 → 2)-β-d-Gal*p*-(1 → 3/4)-d-Glc*p*NAc. The set of potential ligands **1**–**10** with different levels of fucosylation was tested, and their inhibition potency on hemagglutination by PHL was assessed (Table 1, Fig. 2). DMSO-containing blank did not affect the hemagglutination in any way. To assess the contribution of valency to the affinity increase, an affinity improvement factor *β* was calculated as the relationship MIC_basic unit_/(valency × MIC_ligand_).

**Table 1:** Hemagglutination inhibition assay with PHL.

Ligand	Valency	MIC [μM]	Potency_l-Fuc_	*β*_*l-Fuc*_	Potency_Azide1_	*β*_*Azide1*_
Fucoside **2**	1	6,400	0.5	0.5		
**l** **-Fuc**	**1**	**3,200**	**1**	**1**		
Fucoside **4**	1	1,600	2.0	2.0		
Fucoside **3**	1	500	6.4	6.4		
α-Me-Fuc	1	400	7.8	7.8		
**Azide 1**	**1**	**200**	**15.6**	**15.6**	**1**	**1**
Difucoside **5**	2	200	15.6	7.8		
Calixarene **6**	4	100	31.3	7.8	2	0.5
Calixarene **7**	4	100	31.3	7.8	2	0.5
Dendrimer **8**	4	50	62.5	15.6	4	1
Dendrimer **9**	6	25	125	20.8	8	1.3
Dendrimer **10**	12	25	125	10.4	8	0.7

Minimal inhibitory concentrations (MIC) of synthesised inhibitors and their potency towards l-fucose were determined using two independent measurements. To assess the contribution of valency to the MIC, an affinity improvement factor *β* was calculated as the relationship MIC_basic unit_/(valency × MIC_ligand_).

Simple monovalent fucosides **2**–**4** failed to substantially inhibit the hemagglutination caused by PHL, while azide **1** and difucoside **5** were 16-fold better inhibitors than l-fucose and twice as good as methyl α-l-fucopyranoside. If the structures of simple ligands **1**–**5** are compared, it seems that the nonpolar substituent at the anomeric position of l-fucose provides an advantage. The activities of tetravalent calixarenes **6** and **7** are identical, giving a potency of 31.3 without regard to the presence or absence of a *tert*-butyl group on the upper rim of the skeleton. Tetravalent dendrimer **8** was as efficient inhibitor of hemagglutination as calixarenes **6** and **7**, although it occupies a different topology. Hexa- and dodecafucosylated dendrimers **9** and **10** were proven to be the best inhibitors. They were more than 100-fold better ligands than natural l-fucose. However, according to the *β* factor calculated with respect to azide **1** (*β*_*Azide****1***_), all multivalent compounds were comparable with their building unit.

### Surface plasmon resonance

**Figure 3:**
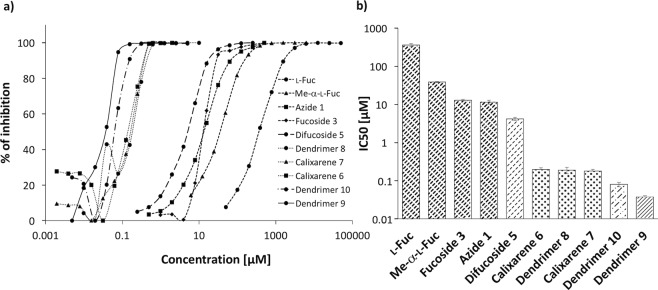
IC_50_ determination of fucoligands towards PHL from SPR measurements. (**a**) A logarithmic plot of inhibition curves calculated from SPR experiments vs. % of inhibition. Individual types of line correspond to monovalent (densely dashed), divalent (dashed), tetravalent (dotted), hexavalent (dash-dotted) and dodecavalent (solid) ligands. (**b**) A logarithmic plot of IC_50_ determined for individual mono/multivalent ligands. Measurements were performed in triplicates and standard deviations were calculated. Individual types of line correspond to monovalent (densely dashed), divalent (dashed), tetravalent (dotted), hexavalent (dash-dotted) and dodecavalent (solid) ligands.

To analyse the competitive inhibition of PHL binding to a multivalent surface, the surface plasmon resonance (SPR) technique was employed. A sensor chip presenting α-l-fucopyranoside residues was treated with a constant concentration of PHL, while an increasing concentration of the ligands was used to determine IC_50_ (Table 2, Fig. 3). Only the ligands comparable with or better than α-Me-Fuc in the hemagglutination assay were investigated by SPR. To assess the contribution of valency to the affinity increase, an affinity improvement factor *β* was calculated as the relationship IC_50,__basic unit_/(valency × IC_50,__ligand_)^30^.

**Table 2:** Inhibition activity of studied ligands towards PHL assessed by SPR.

Ligand	Valency	IC_50_ [μM]	Potency_l-Fuc_	*β*_l*-Fuc*_	Potency_Azide1_	*β*_*Azide1*_
**l** **-Fuc**	**1**	**362** ± **33**	**1**	**1**		
α-Me-Fuc	1	38.5 ± 1.2	9	9		
Fucoside **3**	1	12.9 ± 0.6	28	28		
**Azide 1**	**1**	**11.5** ± **1.4**	**32**	**32**	**1**	**1**
Difucoside **5**	2	4.18 ± 0.42	86	43		
Calixarene **6**	4	0.195 ± 0.023	1,853	464	59	15
Dendrimer **8**	4	0.188 ± 0.037	1,922	481	65	16
Calixarene **7**	4	0.178 ± 0.020	2,029	508	61	15
Dendrimer **10**	12	0.080 ± 0.011	4,538	377	144	12
Dendrimer **9**	6	0.037 ± 0.003	9,762	1,631	311	52

IC_50_ was determined from three independent measurements. To assess the contribution of valency to the IC_50_, an affinity improvement factor *β* was calculated as the relationship IC_50__,__basic unit_/(valency × IC_50__,__ligand_).

Comparing the IC_50_ values and potency, the results from SPR are in good agreement with the finding obtained by hemagglutination. Azide **1** and fucoside **3** exhibit 32-fold and 38-fold, respectively, stronger affinity to PHL than its counterpart l-fucose; in fact, an 86-fold increase in affinity was found for difucoside **5**. The affinity improvement factors of tetravalent calixarenes **6** and **7** as well as dendrimer **8** were comparable with *β*_*Azide****1***_ value reaching approximately 15. The hexavalent dendrimer **9** was the best ligand of the lectin PHL with IC_50_ = 37 nM, *β*_*Azide****1***_ = 52 and with potency 9,800-fold and 310-fold higher than l-fucose and azide **1**, respectively. Although dodecavalent dendrimer **10** was also an efficient ligand (IC_50_ = 80 nM), its affinity improvement factor was substantially lower (*β*_*Azide****1***_ = 12) than that of the hexavalent dendrimer **9**. This observation could be caused by unwanted side effects such as cross-linking or aggregation induced by the sterically demanding structure of **10**. Nevertheless, the data support a strong glycoside clustering effect.

### Isothermal titration calorimetry

**Figure 4:**
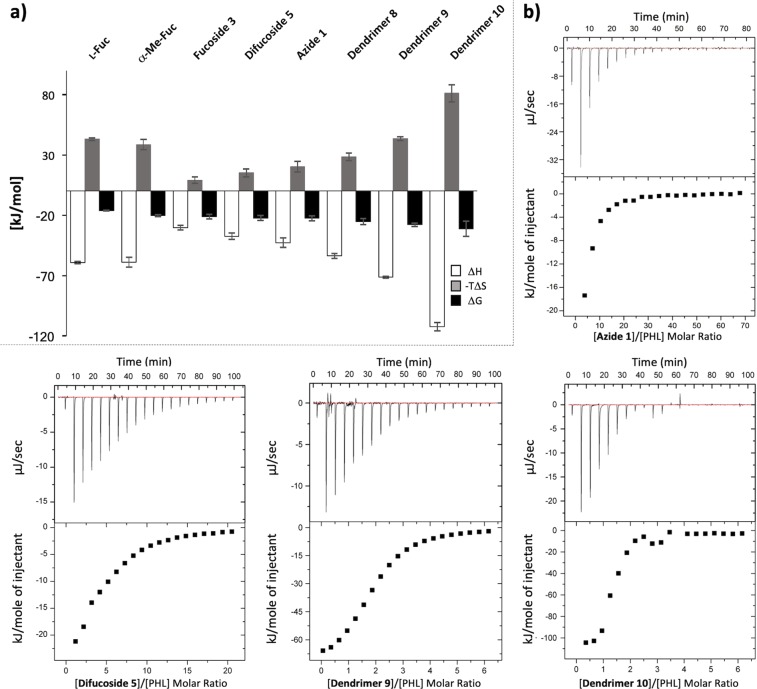
(**a**) Thermodynamic profiles for interaction between PHL and fucoligands determined by isothermal titration calorimetry at 25 °C. (**b**) Selected ITC curves of PHL (50 μM) titration by azide 1 (5 mM), difucoside 5 (5 mM), dendrimer 9 (1.5 mM) and dendrimer 10 (1.5 mM). 20 injections of 2.0 μl of fucoclusters were added every 240 s to a PHL-containing cell. The lower plots show the total heat released as a function of total ligand concentration for the titration shown in the upper panels. Due to low affinity of PHL to monovalent compounds, n, ΔH and ΔS values may suffer from higher inaccuracy.

The binding of PHL to different α-l-fucoside compounds was further characterized by isothermal titration calorimetry (ITC), enabling determination of the complete thermodynamic profile of the molecular interactions (Table 3, Fig. 4). As for calixarenes, both compounds caused a cross-linking/aggregation of PHL accompanied by the formation of visible precipitates. Because of this, it was not possible to evaluate the data, despite the obvious interaction. The dendrimers also cross-linked/aggregated the protein as with calixarenes, however the curves provided clear outcomes with an affinity in the low micromolar range. The dissociation constants (K_D_) of all dendrimers correlate with their valency – the higher number of fucose, the more decreased K_D_. This implies that dodecafucosylated dendrimer **10** was assessed as the best inhibitor, with K_D_ almost 4 times better than the second-best hexavalent dendrimer **9** and 33 times better than azide **1** (*β*_*Azide****1***_ = 3). The stoichiometry value n corresponds to the number of terminal fucoses per ligand molecule. The n value decreases from 4 to 1 as the number of fucose units increases from 1 to 12 (Table 3).

**Table 3:** Thermodynamic profiles for interaction between PHL and ligands determined by isothermal titration calorimetry at 25 °C.

Ligand	Valency	n	K_D_ [μM]	−ΔH (kJ/mol)	−TΔS (kJ/mol)	−ΔG (kJ/mol)	Potency _l-Fuc_	*β*_*l-Fuc*_	Potency_Azide1_	*β*_*Azide1*_
**l** **-Fuc**	**1**	3*	**1,395** ± **33**	**59.5** ± **1.0**	**43.2** ± **1.0**	**16.3** ± **0.4**	**1**	**1**		
α-Me-Fuc	1	2.9 ± 0.2	267 ± 11	59.0 ± 4.2	38.6 ± 4.3	20.4 ± 0.9	5	5		
Fucoside **3**	1	5.4 ± 0.3	187 ± 16	30.3 ± 2.0	9.0 ± 2.6	21.3 ± 1.8	7	7		
Difucoside **5**	2	3.9 ± 0.2	120 ± 11	37.5 ± 2.6	15.1 ± 3.3	22.4 ± 2.1	12	6		
**Azide 1**	1	4.1 ± 0.3	105 ± 9	42.9 ± 3.9	20.2 ± 4.4	22.7 ± 2.0	13	13	**1**	**1**
Dendrimer **8**	4	3.0 ± 0.1	36.9 ± 3.7	53.8 ± 1.9	28.6 ± 3.2	25.3 ± 2.5	38	9	3	1
Dendrimer **9**	6	2.0 ± 0.0	12.5 ± 0.6	71.5 ± 0.8	43.5 ± 1.6	28.0 ± 1.3	112	19	8	1
Dendrimer **10**	12	1.2 ± 0.0	3.2 ± 0.6	112.6 ± 3.5	81.2 ± 7.2	31.4 ± 6.3	436	36	33	3

Thermodynamic parameters were calculated from two independent measurements. To assess the contribution of valency to the affinity increase, an affinity improvement factor *β* was calculated as the relationship K_D,basic unit_/(valency × K_D,ligand_).

*The stoichiometry value of l-fucose was fixed during the fitting procedure because of the low-affinity interaction that also causes that ΔH and ΔS values may suffer from higher inaccuracy. The value was based on the α-Me-Fuc measurement and on the X-ray crystal structure of the PHL/BGH complex.

As for monovalent (azide **1**, fucoside **3**) and divalent (difucoside **5**) compounds, all of them exhibited a low affinity towards PHL (in the millimolar range), as is usually observed for lectin/saccharide interactions. They also correspond with the same *β* factor.

### *P. asymbiotica* cross-linking – Bacterial aggregation properties

**Figure 5:**
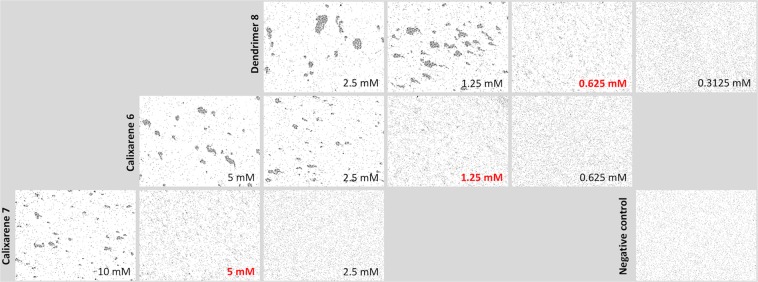
Representative images of optical microscopy observation (200x) of bacterial cell aggregation for *Photorhabdus asymbiotica* subsp*. australis* with different fucosylated clusters. Bacterial cells in PBS buffer were used as a negative control, shown in the bottom right image. Two-fold serially diluted carbohydrate compounds **6**, **7** and **8** were able to agglutinate the cells at minimal concentrations of 1.25 mM, 5 mM and 0.625 mM, respectively. The background of all pictures was subtracted in the software GIMP.

Because of the aggregation of PHL by calixarenes and dendrimers during SPR and ITC experiments, we decided to compare tetravalent compounds at a higher level. We performed a series of *in vitro* aggregation assays to reveal their aggregation properties towards *P. asymbiotica* subsp*. australis* (Table 4, Fig. 5). Prior to each assay, we confirmed the absence of aggregates in two negative controls – bacterial cells in PBS buffer and in the presence of azide **1** as a monomer. With all chosen compounds, we observed bacterial aggregates of a variable size that confirmed that they are capable of interacting with a cell surface. This effect may be caused by interaction of the multivalent inhibitors with PHL or other receptors. The concentration of the first appearance of aggregates varied according to the nature of the ligand used. The highest efficiency (0.625 mM) was observed for the tetravalent dendrimer **8**, as we expected. Calixarene **6** proved to be only half as good as dendrimer **8**, and aggregated cells at a concentration of 1.25 mM. An unexpected observation was that calixarene **7** had the lowest aggregation potential. In contrast to other techniques (hemagglutination, SPR and ITC), where calixarenes **6** and **7** provided similar results, during cross-linking experiments calixarene **7** was four times worse than calixarene **6**. At the same time, both tetrameric calixarenes **6** and **7** are in a 1,3-*alternate* conformation with the two-faced presentation of the fucopyranosyl epitopes on opposite regions probably occupying a similar spatial orientation. The only difference is the *tert*-Bu group on the upper rim in calixarene **6**, which might contribute to nonpolar interactions with whole *P. asymbiotica* cells.

**Table 4:** Determination of minimal concentration of multivalent fucoclusters able to aggregate *P. asymbiotica* subsp*. australis* cells.

Ligand	Valency	The lowest concentration able to aggregate *P. asymbiotica* cells [mM]
Azide **1**	1	No visible aggregation in used concentrations
Calixarene **7**	4	5
Calixarene **6**	4	1.25
Dendrimer **8**	4	0.625

Three independent measurements were performed.

### X-ray structure of PHL/fucosides complex

The PHL lectin forms a seven-bladed β-propeller assembling into a homo-dimer. Both units contain two types of binding sites situated between the blades and have different amino acid compositions and binding preferences^16^. As mixing PHL with the branched compounds leads to protein precipitation and thus prevents co-crystallization, the direct study of complexes was not possible. Soaking ligand-free PHL crystals with a low concentration of individual branched compounds did not result in complex formation while soaking with their higher concentrations led to a crystal decomposition. Therefore, we soaked the PHL crystals with monomeric fucoside **2**, **3** and **4**, respectively. The structure of these complexes was determined using X-ray diffraction (Table 5).

**Table 5:** Data collection and refinement statistics for PHL complexes.

Molecule	PHL/fucoside 2	PHL/fucoside 3	PHL/fucoside 4
PDB ID	6FHX	6FHY	6FLU
***Data collection***			
Beamline	BESSY II 14.3	BESSY II 14.3	BESSY II 14.3
Wavelength (Ǻ)	0.8943	0.8943	0.8943
Space group	P3_2_21	P3_2_21	P3_2_21
**Unit-cell parameters**			
a and b (Å)	81.57	81.35	81.94
c (Å)	222.91	222.78	224.65
α = β [°]	90	90	90
γ [°]	120	120	120
Resolution range (Ǻ)	44.58-2.34 (2.40-2.34)	44.56-1.86 (1.91-1.86)	44.93-1.78 (1.82-1.78)
Reflections measured	407636 (58160)	813765 (116126)	941162 (132122)
Unique reflections	37127 (5331)	73161 (10443)	85234 (12197)
Completeness (%)	99.9 (99.2)	99.9 (99.2)	100.0 (99.5)
CC1/2	99.2 (68.9)	99.6 (57.6)	99.1 (53.0)
R_merge_^†^	0.218 (0.989)	0.193 (1.383)	0.242 (1.325)
<I/σ (I)>	12.0 (2.7)	12.5 (1.8)	9.0 (1.7)
Multiplicity	11.0 (10.9)	11.1 (11.1)	11.0 (10.8)
Wilson B factor (Ǻ^−2^)	19.95	16.80	8.14
***Refinement***			
Reflections used	35215 (2533)	69572 (5027)	80956 (5879)
Reflections used for R_free_	1850 (150)	3505 (254)	4196 (293)
R factor^‡^ (%)	19.5 (26.4)	18.1 (28.7)	18.4 (28.7)
R_free_^‡^ (%)	25.2 (32.4)	21.5 (32.0)	22.2 (30.4)
R.m.s.d. bond lengths (Ǻ)	0.013	0.009	0.019
R.m.s.d. bond angles (°)	1.650	1.466	1.712
R.m.s.d. chirals (Ǻ^3^)	0.101	0.085	0.113
No. of non-H atoms (total)	5736	6042	6324
No. of water molecules	238	441	700
No. of ligand atoms	165	241	216
No. of ions	3	1	9
Mean B factor (Ǻ^2^)			
Non-H atoms	25.04	21.20	13.17
Water molecules	22.99	28.32	20.72
Ligands	39.12	35.44	24.64
Ions	32.40	36.76	23.78
Ramachandran plot (%)			
Residues in most favourable regions	95.8	97.2	96.7
Residues in allowed regions	4.2	2.8	3.3
Rotamer outliers (%)	3.2	3.2	3.5

Values in parentheses correspond to the highest resolution shell.

^†^R_merge_ = ∑│I_i_ − < I > │/∑I_i_, where I_i_ is the intensity of observation and < I > is the mean value for that reflection.

^‡^R factor = ∑ ││F_o_(h)│ − │F_c_(h)││/∑_h_ F_o_(h), where F_o_ and F_c_ are the observed and calculated structure-factor amplitudes, respectively.

**Figure 6:**
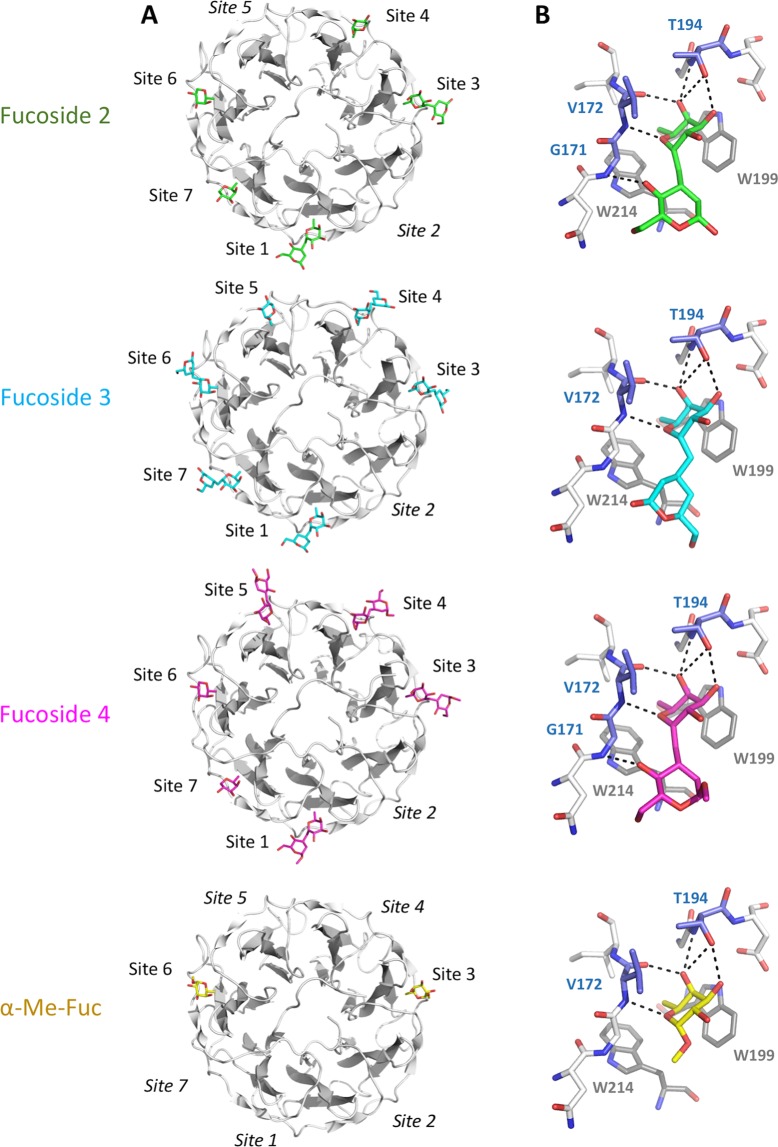
Structure of PHL with fucosides **2**–**4** and α-Me-Fuc from 5MXE structure for comparison. (**A**) Overall structure of chain A of PHL complexes. Individual binding sites labelled, label in italics for empty site. Fucosides shown as sticks. (**B**) Binding site 3 with coordinated ligand. Key residues labelled. Colour code: fucoside **2** – green, fucoside **3** – cyan, fucoside **4** – magenta, α-Me-Fuc – yellow, CH-π interacting tryptophans – grey, H-bond interacting residues – blue.

Crystal structures confirmed that all the studied fucosides were able to bind the lectin PHL. In each complex, the electron density was clear enough to determine the position of the fucosyl part of the compounds in 6 out of 7 fucose-binding sites of at least one PHL monomer unit – A or B (Table 6, Fig. 6), while site 2 is occupied by Met60 of the neighbouring monomer, as was observed previously^16^. This demonstrates the binding potency of studied fucosides and experimentally confirmed a presence of at least 6 binding sites per monomer. In previous studies, only 2 to 5 binding sites were detected^16,31^. The second saccharide unit was clearly assigned for all three fucosides **2**–**4** in sites 1 and 3. For fucoside **3** and **4**, the second saccharide was also distinguishable in sites 4-7 of at least one protein monomer (Table 6). This allows us to compare the binding mode for *C*-glycoside-based molecules and published previously *O*-glycoside structures. The fucose moiety is coordinated in the same way as was shown for α-Me-Fuc and blood group H trisaccharide^16^. Briefly, the O3, O4 and O5 of fucose are coordinated by the backbone atoms of two neighbouring loops and in sites 1, 3, and 5–7 also by the side chain of nearby threonine. The C6 methyl group and nonpolar surface of fucose are stabilized by a CH-π interaction with two tryptophan side chains. Considering fucosides **2**–**4**, the position of the bridging methylene group is close to the α-Me-Fuc O1 atom, which is not directly coordinated by the protein in the complex. Additional direct interactions between the protein and the studied molecules are infrequent. The O4 of the d-*arabino*-hexopyranosyl unit in fucoside **2** and **4** is coordinated by a glycine backbone nitrogen in sites 1, 3, 5, and 6, while the O6 atom of fucoside **3** is linked to a tryptophan backbone oxygen in sites 4 and 6. When comparing the overall orientation of the second hexopyranosyl unit of fucosides **2**–**4**, it is similar to the galactose unit of blood group H trisaccharide, with minor distortions in individual binding sites influenced e.g. by crystal packing. The remaining parts of fucosides **2** and **3** were not resolved in the electron density, suggesting their negligible importance for ligand binding.

**Table 6:** Binding site occupancy of PHL complexes with fucosides **2**–**4**.

Molecule	PHL/fucoside 2		PHL/fucoside 3		PHL/fucoside 4	
Monomer unit	A	B	A	B	A	B
Site 1	FA	FA	FA	FA	FA	FA
Site 2	Met60	Met60	Met60	Met60	Met60	Met60
Site 3	FA	FA	FA	FA	FA	FA
Site 4	F	F	FA	FA	FA	F
Site 5	—	FA	F	FA	FA	F
Site 6	F	F	FA	FA	F	FA
Site 7	F	F	FA	FA	F	FA

F – fucosyl moiety, A – d-*arabino*-hexopyranosyl moiety, Met60 – Met60 from symmetry-related PHL molecule.

## Conclusions

In the previous study^16^, we identified an interesting lectin from emerging human pathogen *P. asymbiotica* harbouring seven potential fucose-binding sites per monomer and able to modulate innate immune system of human. Infection strategies used by pathogens often involve highly specific protein/carbohydrate interactions and therefore design of suitable inhibitors preventing this interaction is needed. Thus, we tested inhibition potency of l-fucose-based compounds with different valences and topologies. All these structures share the same feature of bearing *C*-glycosidic bond instead of the common but physiologically unstable *O*-glycosidic bond.

α-l-fucopyranosyl-containing mono-, di-, tetra-, hexa- and dodecavalent ligands were investigated by hemagglutination, ITC, SPR, X-ray crystallography, and cell cross-linking. The binding mode of monovalent ligands was studied via crystal complexes with PHL where the electron density proved the presence of the fucosyl part of the compounds in all accessible fucose-binding sites. The second saccharide unit of studied fucosides was only marginally coordinated by PHL binding sites what, together with affinity comparable to monosaccharides, suggests a low importance of this type of compounds for inhibitor design. On the other hand, the affinity towards azide **1** is thirteen times higher than towards l-fucose and comparable to previously studied propargyl-α-l-fucoside^31^. This suggests, together with data from the previously studied PHL complexes, that introduction of a linker with delocalized π-electrons has not negligible contributory effect to binding affinity and increases also binding site availability/occupancy (4–5 binding sites for azide **1**/propargyl-α-l-fucoside vs. 2–3 sites for α-Me-Fuc). This effect was already reported for the PA-IIL lectin and *p*-nitrophenyl-α-l-fucoside^32^. The affinity of PHL for multivalent ligands reached low micromolar values, which corresponds to an affinity three orders of magnitude higher than that of standard l-fucose and two orders of magnitude higher than that of monovalent azide **1**. We have further demonstrated that all of the tested compounds were able to inhibit the PHL binding towards both artificial and natural fucosylated surfaces. Generally, the potency of ligands depended on the valency and types of polyvalent structures (calix[4]arenes and dendrimers). Even though the relation between the increasing number of fucoses per cluster and increasing affinity, the most active compound was a hexavalent dendrimer exhibited an IC_50_ of 37 nM and K_D_ of 13 µM. Several tetravalent compounds of different type were also tested for their ability to agglutinate *P. asymbiotica* bacteria. The cell clumps at different concentrations of tested compounds were observed further supporting the previously reported presence of lectins on surface as was also proved for other bacteria (e.g. *P. aeruginosa*, *E. coli*)^31,33–35^.

Based on the findings presented in this work, we consider these compounds as a milestone on the way to design efficient inhibitors targeting *P. asymbiotica*.

## Methods

### Ligand preparation

All ligands were prepared as 50 mM stock solutions. Lyophilized azide 1, fucosides 2–4, difucoside 5, and dendrimers 8–10 were dissolved in the deionized water, whereas calixarenes 6–7 were dissolved in DMSO.

### Protein production and purification

The PHL lectin was produced in *Escherichia coli* Tuner(DE3)/pET25b_*phl* cells as previously published^16^. In short, the cells were grown in LB broth medium containing 100 μM ampicillin at 37 °C. After reaching an OD_600_ of 0.5, gene expression was induced with 0.2 mM isopropyl β-d-1-thiogalactopyranoside (IPTG). Cells were incubated for an additional 20 hours at 18 °C, harvested by centrifugation at 12,000 g for 10 min and resuspended in buffer A (20 mM Tris/HCl, 300 mM NaCl, pH 7.5). Harvested cells were stored at −20 °C prior to protein purification.

For protein purification, cells were disintegrated by sonication (VCX 500, Sonics & Materials, Inc., USA) and the cytosolic fraction containing soluble PHL was collected by centrifugation at 21,000 g at 4 °C for 1 hour and filtrated through a 0.45 μm pore size filter (Carl Roth, Germany). Recombinant protein PHL was purified with isocratic elution on a d-mannose-agarose (Sigma-Aldrich, USA) resin equilibrated with buffer A by affinity chromatography using an ÄKTA FPLC system (GE Healthcare, UK) and used for further studies. For agglutination assays, purified PHL was dialyzed against PBS buffer (137 mM NaCl, 2.7 mM KCl, 8 mM Na_2_HPO_4_, 1.47 mM KH_2_PO_4_, pH 7.4).

### Hemagglutination inhibition assay

Human red blood cells (RBCs) O were processed according to previously published work^16^. In brief, RBCs O treated with sodium citrate were washed four times by PBS buffer (137 mM NaCl, 2.7 mM KCl, 8 mM Na_2_HPO_4_, 1.47 mM KH_2_PO_4_, pH 7.4), diluted to 50% by PBS with 0.005% (w/w) sodium azide and treated by 0.1% papain for the duration of 30 minutes.

Hemagglutination inhibition assay was performed for specificity and semi-quantitative affinity of the PHL interaction with the compounds. All carbohydrate inhibitors in 50 mM starting concentration were serially diluted in the PBS buffer and used for a determination of the lowest inhibiting concentration. As a blank, the PBS buffer supplemented with the appropriate amount of DMSO was used in order to exclude potential interference with the interaction. The individual samples were mixed with the lectin in concentration 2 mg/ml in a 5 μl:5 μl ratio. Thereafter, 10 μl of 10% RBCs O in PBS buffer was added, thoroughly mixed and incubated for 10 minutes at room temperature^36^. Following incubation, the mixture was again mixed and the reaction was observed on microscope slides using Levenhuk 2L NG microscope with Levenhuk D2L digital camera (Levenhuk, USA). Imagines were obtained via software ToupView for Windows (Levenhuk). The positive (PHL without inhibitor) and negative control (reaction without PHL) were prepared and processed in the same way using the appropriate volume of PBS buffer instead of the not included components. The lowest concentration of inhibitor able to inhibit agglutination was determined and compared with the standard (l-fucose). Minimal inhibitory concentrations (MIC) of synthesised inhibitors were determined from two independent measurements.

### Surface plasmon resonance

Surface plasmon resonance (SPR) experiments were performed on a BIAcore T200 instrument (GE Healthcare, UK) at 25 °C, using buffer A supplemented with 0.05% Tween 20 as a running buffer. α-l-fucoside was immobilized onto CM5 sensor chip (GE Healthcare, UK) covered with a carboxymethylated dextran matrix. The sensor chip surface was activated with *N*-ethyl-*N*-(3-dimethylaminopropyl)carbodiimide/*N*-hydroxysuccinimide solution and then coated with streptavidin using the manufacturer’s standard protocol using HBS buffer (10 mM HEPES, 150 mM NaCl, 0.05% Tween 20, pH 7. 5). Unreacted groups were blocked with 1 M ethanolamine-HCl, pH 8.5. Biotinylated carbohydrate (biotinilated monomeric probes, Lectinity, Russia) was injected onto particular measuring channel and pure biotin on blank channel at a flow rate of 5 μl/min.

SPR inhibition measurements were carried out on measuring channel with l-fucoside at a flow rate of 5 µl/min. PHL was diluted to a concentration of 20 μg/ml by the buffer A supplemented with 0.05% Tween 20, mixed 1:1 (v/v) with various concentrations of inhibitors (500–5 μM) in the same buffer and injected onto the sensor chip. The response of lectin bound to the sugar surface at equilibrium was plotted against the concentration of inhibitor in order to determine IC_50_ (concentration of inhibitor resulting in 50% inhibition of binding). As IC_50_ is not a constant and depends on the experimental set-up, a parameter called potency was used for characterization. The potency of a tested inhibitor is the ratio of IC_50_ of a chosen standard inhibitor (in this case l-fucose) and the inhibitor in question. Pure PHL lectin was used as a control (0% inhibition) and channel with pure biotin served as a blank. IC_50_ was determined from triplicates.

### Isothermal titration calorimetry

PHL protein in buffer A was equilibrated at room temperature at least for 30 min before ITC measurement. All ITC experiments were performed using ITC200 calorimeter (Malvern Panalytical, UK) at 25 °C. Carbohydrate ligands dissolved in buffer A were used at different concentrations. Protein in the calorimeter cell (50 μM) was titrated by consecutive additions (2 μl) of the ligand (1.5–5 mM) in the syringe while stirring at 1000 rpm. Integrated heat effects were analysed by nonlinear regression using a single-site binding model and global fitting in Origin 7 software (Microcal Instruments)^37^. The thermodynamic parameters of synthesised inhibitors were determined from two independent measurements.

### Crystallization and data collection

PHL was concentrated to 13.6 mg/ml using an ultrafiltration unit with a 10-kDa cut-off membrane (Vivaspin 20, Sartorius, Germany) and crystallized under previously described conditions^16^. The crystals were obtained under the following conditions: 4 μl sitting drop, protein solution mixed with precipitant (3.7 M NaCl, 100 mM Hepes, pH 7.5) in ratios 1:1, 3:5, 1:3 and 1:7. The drops were set against 0.5 ml of the same equilibration precipitant. To determine the PHL structure complexed with fucosides **2**–**4**, the crystals of PHL were soaked in a 10 mM solution of compounds **2**–**4** for 1 hour. The crystals were cryo-protected using 40% PEG 400 and frozen in liquid nitrogen. The diffraction data of PHL complexed with saccharides were collected at the BESSY II electron storage ring (Berlin-Adlershof, Germany)^38^.

### Structure determination

Images were processed using XDSAPP^39^ and converted to structure factors using the program package CCP4 v.6.5^40^, with 5% of the data reserved for R_free_ calculation. The structures of PHL complexes were solved by molecular replacement with Phaser^41^ using the monomeric coordinates of the ligand-free PHL structure (5MXE). Refinement of the molecule was performed using REFMAC5^42^ alternated with manual model building in Coot v.0.8^43^. Sugar-derived ligand coordinates were established using JLigand^44^ included in CCP4 program package. Ligands and other compounds that were present were placed manually using Coot. Water molecules were added by Coot and checked manually. The addition of alternative conformations, where necessary, resulted in final structures that were validated using the MolProbity^45^ (http://molprobity.biochem.duke.edu) validation server and were deposited in the PDB as entries 6FHX, 6FHY and 6FLU. Molecular drawings were prepared using Pymol (Schrödinger, Inc.).

### *P. asymbiotica* growth

*P. asymbiotica* subsp*. australis* was inoculated from stock cells stored at −80 °C into a standard LB media and were grown in 30 °C under an orbital shaking overnight. Bacterial growth was tracked by measuring the optical density of the broth at 600 nm (OD_600_) using a spectrophotometer (Biochrom Ltd., BioPhotometer WGA CO 8000 Biowave). Cells were harvested when reaching an OD_600_ = 0.5. The culture was centrifuged at 4,300 g and room temperature for 2 minutes (Minispin, Eppendorf, Germany) and cells were then washed three times with PBS buffer. Cells were diluted in PBS to OD600 of 2.0 and kept in a fridge until performing the aggregation assay.

### Cell aggregation assay

*P. asymbiotica* subsp*. australis* cells and fucosylated compounds in a concentration ranging from 50 mM to 3.125 mM were gently mixed in a 9:1 (v/v) ratio. The reaction mixtures were left at room temperature for 10 minutes. 5 µl of reaction mixture was then transferred onto a microscope slide and observed under a fluorescence microscope (OLYMPUS IX81 Microscope IX81F-3 with IX2-UCB-2 Controller and X-Cite 120PC Q; Olympus and Excelitas Technologies, Japan, resp. USA). As a negative control, bacterial cells with/without azide **1** as a monomer were used. Three independent measurements were performed.

## References

[1] Varki A (2017). Biological roles of glycans. Glycobiology.

[2] Sharon N (2006). Carbohydrates as future anti-adhesion drugs for infectious diseases. Biochim. Biophys. Acta.

[3] Imberty A, Wimmerová M, Mitchell EP, Gilboa-Garber N (2004). Structures of the lectins from *Pseudomonas aeruginosa*: insight into the molecular basis for host glycan recognition. Microbes Infect..

[4] Farmer JJ (1989). *Xenorhabdus luminescens* (DNA hybridization group 5) from human clinical specimens. J. Clin. Microbiol..

[5] Peel MM (1999). Isolation, identification, and molecular characterization of strains of *Photorhabdus luminescens* from infected humans in Australia. J. Clin. Microbiol..

[6] Mulley G (2015). From insect to man: *Photorhabdus* sheds light on the emergence of human pathogenicity. PloS One.

[7] Gerrard JG (2006). Nematode symbiont for *Photorhabdus asymbiotica*. Emerg. Infect. Dis..

[8] Peat SM (2010). A robust phylogenetic framework for the bacterial genus *Photorhabdus* and its use in studying the evolution and maintenance of bioluminescence: A case for 16S, gyrB, and glnA. Mol. Phylogenet. Evol..

[9] Kuwata R, Yoshiga T, Yoshida M, Kondo E (2008). Mutualistic association of *Photorhabdus asymbiotica* with Japanese heterorhabditid entomopathogenic nematodes. Microbes Infect..

[10] Thanwisai A (2012). Diversity of *Xenorhabdus* and *Photorhabdus* spp. and their symbiotic entomopathogenic nematodes from Thailand. PLoS ONE.

[11] Gerrard JG, McNevin S, Alfredson D, Forgan-Smith R, Fraser N (2003). *Photorhabdus* species: bioluminescent bacteria as emerging human pathogens?. Emerg. Infect. Dis..

[12] Gerrard J, Waterfield N, Vohra R, ffrench-Constant R (2004). Human infection with *Photorhabdus asymbiotica*: an emerging bacterial pathogen. Microbes Infect..

[13] Weissfeld AS (2005). *Photorhabdus asymbiotica*, a pathogen emerging on two continents that proves that there is no substitute for a well-trained clinical microbiologist. J. Clin. Microbiol..

[14] Costa SCP (2010). Recent insight into the pathogenicity mechanisms of the emergent pathogen *Photorhabdus asymbiotica*. Microbes Infect..

[15] Machado, R. A. R. *et al*. Whole-genome-based revisit of *Photorhabdus phylogeny*: proposal for the elevation of most *Photorhabdus* subspecies to the species level and description of one novel species *Photorhabdus bodei* sp. nov., and one novel subspecies *Photorhabdus laumondii* subsp. *clarkei* subsp. nov. *Int. J. Syst. Evol. Microbiol*. **68**, 2664–2681 (2018). 10.1099/ijsem.0.002820 29877789

[16] Jančaříková G (2017). Characterization of novel bangle lectin from *Photorhabdus asymbiotica* with dual sugar-binding specificity and its effect on host immunity. PLOS Pathog..

[17] Chemani C (2009). Role of LecA and LecB lectins in *Pseudomonas aeruginosa* - induced lung injury and effect of carbohydrate ligands. Infect. Immun..

[18] Wu XR, Sun TT, Medina JJ (1996). *In vitro* binding of type 1-fimbriated *Escherichia coli* to uroplakins Ia and Ib: relation to urinary tract infections. Proc. Natl. Acad. Sci. USA.

[19] Kumar A (2016). A novel fucose-binding lectin from *Photorhabdus luminescens* (PLL) with an unusual hepta-bladed β-propeller tetrameric structure. J. Biol. Chem..

[20] Beshr G (2017). *Photorhabdus luminescens* lectin A (PllA): A new probe for detecting α-galactoside–terminating glycoconjugates. J. Biol. Chem..

[21] Diggle SP (2006). The galactophilic lectin, LecA, contributes to biofilm development in *Pseudomonas aeruginosa*. Environ. Microbiol..

[22] Bertolotti B (2017). Polyvalent C-glycomimetics based on l-fucose or d-mannose as potent DC-SIGN antagonists. Org. Biomol. Chem..

[23] Bertolotti B (2016). Nonhydrolyzable C-disaccharides, a new class of DC-SIGN ligands. Carbohydr. Res..

[24] Kašáková M (2018). Selectivity of original C-hexopyranosyl calix[4]arene conjugates towards lectins of different origin. Carbohydr. Res..

[25] Rostovtsev VV, Green LG, Fokin VV, Sharpless KB (2002). A Stepwise Huisgen Cycloaddition Process: Copper(I)-Catalyzed Regioselective “Ligation” of Azides and Terminal Alkynes. Angew. Chem. Int. Ed..

[26] Tornøe CW, Christensen C, Meldal M (2002). Peptidotriazoles on Solid Phase: [1,2,3]-Triazoles by Regiospecific Copper(I)-Catalyzed 1,3-Dipolar Cycloadditions of Terminal Alkynes to Azides. J. Org. Chem..

[27] Lundquist JJ, Toone EJ (2002). The cluster glycoside effect. Chem. Rev..

[28] Cecioni S, Imberty A, Vidal S (2015). Glycomimetics versus multivalent glycoconjugates for the design of high affinity lectin ligands. Chem. Rev..

[29] Bernardi A (2013). Multivalent glycoconjugates as anti-pathogenic agents. Chem. Soc. Rev..

[30] Tabarani G (2009). DC-SIGN neck domain is a pH-sensor controlling oligomerization: SAXS and hydrodynamic studies of extracellular domain. J. Biol. Chem..

[31] Jančaříková, G. *et al*. Synthesis of α-L-fucopyranoside-presenting glycoclusters and investigation of their interaction with recombinant *Photorhabdus asymbiotica* lectin (PHL). *Chem. - Eur. J*., 10.1002/chem.201705853 (2018). 10.1002/chem.201705853 29341313

[32] Mitchell EP (2004). High affinity fucose binding of *Pseudomonas aeruginosa* lectin PA-IIL: 1.0 Å resolution crystal structure of the complex combined with thermodynamics and computational chemistry approaches. Proteins Struct. Funct. Bioinforma..

[33] Boukerb AM (2014). Antiadhesive properties of glycoclusters against *Pseudomonas aeruginosa* lung infection. J. Med. Chem..

[34] Yu G (2013). A Sugar-functionalized amphiphilic pillar[5]arene: Synthesis, self-assembly in water, and application in bacterial cell agglutination. J. Am. Chem. Soc..

[35] Gestwicki JE, Strong LE, Cairo CW, Boehm FJ, Kiessling LL (2002). Cell aggregation by scaffolded receptor clusters. Chem. Biol..

[36] Adamová L, Malinovská L, Wimmerová M (2014). New sensitive detection method for lectin hemagglutination using microscopy. Microsc. Res. Tech..

[37] Wiseman T, Williston S, Brandts JF, Lin L-N (1989). Rapid measurement of binding constants and heats of binding using a new titration calorimeter. Anal. Biochem..

[38] Mueller U (2012). Facilities for macromolecular crystallography at the Helmholtz-Zentrum Berlin. J. Synchrotron Radiat..

[39] Krug M, Weiss MS, Heinemann U, Mueller U (2012). *XDSAPP*: a graphical user interface for the convenient processing of diffraction data using *XDS*. J. Appl. Crystallogr..

[40] Winn MD (2011). Overview of the *CCP* 4 suite and current developments. Acta Crystallogr. D Biol. Crystallogr..

[41] McCoy AJ (2007). Phaser crystallographic software. J. Appl. Crystallogr..

[42] Murshudov GN (2011). *REFMAC* 5 for the refinement of macromolecular crystal structures. Acta Crystallogr. D Biol. Crystallogr..

[43] Emsley P, Lohkamp B, Scott WG, Cowtan K (2010). Features and development of Coot. Acta Crystallogr. D Biol. Crystallogr..

[44] Lebedev AA (2012). JLigand: a graphical tool for the CCP4 template-restraint library. Acta Crystallogr. D Biol. Crystallogr..

[45] Chen VB (2010). *MolProbity*: all-atom structure validation for macromolecular crystallography. Acta Crystallogr. D Biol. Crystallogr..

